# Improved healing of critical-size femoral defect in osteoporosis rat models using 3D elastin/polycaprolactone/nHA scaffold in combination with mesenchymal stem cells

**DOI:** 10.1007/s10856-021-06495-w

**Published:** 2021-03-08

**Authors:** Fatemeh Hejazi, Vahid Ebrahimi, Mehrdad Asgary, Abbas Piryaei, Mohammad Javad Fridoni, Ali Asghar Kermani, Fatemeh Zare, Mohammad-Amin Abdollahifar

**Affiliations:** 1 Department of Advanced Technology, Shiraz University, Shiraz, Iran; 2 Department of Anatomy and Cell Biology, School of Medicine, Mashhad University of Medical Sciences, Mashhad, Iran; 3 Department of Biology and Anatomical Sciences, School of Medicine, ShahidBeheshti University of Medical Sciences, Tehran, Iran; 4 Urogenital Stem Cell Research Center, Shahid Beheshti University of Medical Sciences, Tehran, Iran; 5 Department of Stem Cells and Developmental Biology, Cell Science Research Center, Royan Institute for Stem Cell Biology and Technology, ACECR, Tehran, Iran, Tehran, Iran; 6 Department of Anatomical Sciences, School of Medicine, Zanjan University of Medical Sciences, Zanjan, Iran; 7 Department of Molecular, Cellular and Developmental Biology, University of Michigan, Ann Arbor, MI 48109 USA

## Abstract

Osteoporosis is a common bone disease that results in elevated risk of fracture, and delayed bone healing and impaired bone regeneration are implicated by this disease. In this study, Elastin/Polycaprolactone/nHA nanofibrous scaffold in combination with mesenchymal stem cells were used to regenerate bone defects. Cytotoxicity, cytocompatibility and cellular morphology were evaluated in vitro and observations revealed that an appropriate environment for cellular attachment, growth, migration, and proliferation is provided by this scaffold. At 3 months following ovariectomy (OVX), the rats were used as animal models with an induced critical size defect in the femur to evaluate the therapeutic potential of osteogenic differentiation of bone marrow mesenchymal stem cells (BM-MSCs) seeded on 3 dimension (3D) scaffolds. In this experimental study, 24 female Wistar rats were equally divided into three groups: Control, scaffold (non-seeded BM-MSC), and scaffold + cell (seeded BM-MSC) groups. 30 days after surgery, the right femur was removed, and underwent a stereological analysis and RNA extraction in order to examine the expression of *Bmp-2* and *Vegf* genes. The results showed a significant increase in stereological parameters and expression of *Bmp-2* and *Vegf* in scaffold and scaffold + cell groups compared to the control rats. The present study suggests that the use of the 3D Elastin/Polycaprolactone (PCL)/Nano hydroxyapatite (nHA) scaffold in combination with MSCs may improve the fracture regeneration and accelerates bone healing at the osteotomy site in rats.

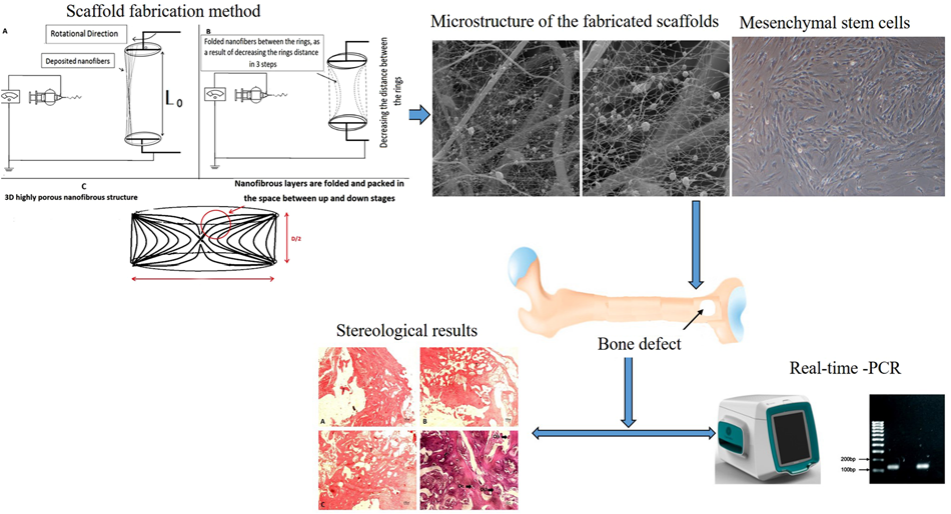

## Introduction

Designing novel biomaterials for tissue engineering purpose is an obvious demand considering ever increasing need for appropriate biocompatibilities and properties to achieve the maximum regeneration [1]. Natural macromolecules which are dominantly present in a number of native tissues, together with synthetic biopolymers and bioactive molecules can provide favorable composition for designing a biomimetic tissue engineering scaffolds. Among the highly potent extracellular matrix (ECM) proteins, structural stability, elastic resilience, and bioactivity of “elastin”, combined with its capacity for self-assembly, make this protein a highly desirable candidate for the fabrication of biomaterials [2].

For effective bone tissue engineering it is essential that the scaffold matrix supports the growth of cells, from division through to maturation, without causing deleterious changes to cell morphology or behavior. To achieve the necessary support infrastructure (physical and biological) for the desired bone regenerative responses, nHA may be used alone or in combination with other materials. nHA also would produce signals that drive the desired cell behavior and activate the activity of bone biomarkers [3].

Osteoporosis is a global age-related disorder described by an imbalance between osteoblasts-associated bone formation and osteoclasts-associated bone resorption, usually as a result of postmenopausal deficiency of estrogen [4, 5]. This condition leads to trabecular and cortical bone mass reduction that subsequently causes several complications such as skeletal instability and fractures [5, 6]. Microarchitecture of the bone structure is altered following bone defects specially osteoporosis [6]. These changes describe the microscopic structure and morphology of trabecular and cortical bones that can be identified using bone histomorphometry and computed tomography (CT) scanning techniques [6, 7].

During normal bone remodeling and healing many growth factors are attributed to induce normal function of osteoblasts, osteocytes and osteoclasts. The ECM and MSCs release growth factors such as bone morphogenic proteins (BMP) and insulin-like growth-factor 1 (IGF-1), platelet-derived growth factor (PDGF), vascular endothelial growth factor (VEGF), fibroblast growth factor-2 (FGF-2). All of these growth factors play critical roles in bone and cartilage development, and they have the ability to trigger proliferation and differentiation of osteoprogenitor cells [8–10], along with activation of osteoblasts to begin collagen type^1^ synthesis [11, 12].

Current treatments for osteoporosis including bone reabsorption inhibitors have several side effects [13, 14]. There is still challenge toward therapeutic approach of osteoporosis [15].

Electrospinning is an outstanding technique for the fabrication of tissue engineering which is capable of producing non-woven fibrous structures with dimensional constituents similar to those of ECM fibers. Apart from the clear advantages and extensive uses, 2D structure produced by conventional electrospinning encounters some practical limitations, such as poor cellular infiltration and ingrowth, non-applicable for 3D defects, poor water absorption, etc. Various attempts have been made to overcome these drawbacks such as electrospinning in wet media, using sacrificial components, manipulating collector design [16]. In the present study, a novel method for the fabrication of three-dimensional scaffold is introduced using electrospinning technique as the base method and an innovative collector for the nanofiber collection.

Considerable attention has been paid to the routine treatments of osteoporosis-related bone fractures using medications. And fewer investigations have been done on the therapeutic effects of local implantation of bio-scaffolds and MSCs during regeneration of osteoporotic defects [17, 18]. Nowadays, tissue engineering has provided new ways for engineering biomaterials from macromolecular size down to molecular-scale accuracy. The resulting biomaterials are predicted to provide advanced technologies in nanomedicine applications [17, 18]. Osteoblasts, osteocytes, and osteoclasts are regarded as basic multicellular units that regulate bone remodeling. Having estrogen receptors enables osteoclasts to elicit protein synthesis by estrogen stimulations [19].

Key requirements of bone regenerative substitutes (especially in the presence of osteoporosis) consist of stimulation of bone growth (osteoinduction) and the presence of a micronetwork of pores and channels to allow cellular migration, transport of nutrients, and gaseous exchange. The composition and geometry of a scaffold should promote the development of new blood vessels and facilitate the passage of the molecular signals that drive growth and repair [3, 20].

In the present study, a combination of highly potent biomolecules which could intensely stimulate the bone regeneration together with a novel method for the fabrication of three-dimensional scaffold is introduced. Electrospinning technique was applied as the base method and an innovative collector was considered for the nanofiber collection. Consequently, highly porous nanofibrous structure is developed in 3D pattern which is suitable for 3D critical size defects. Combination of polycaprolactone, elastin and nano hydroxyapatite was used to prepare the scaffold which provides an appropriate condition for bone tissue regeneration. Structural and physical evaluations together with in vitro and in vivo analysis were performed to examine the scaffolds performance. In vitro tests were done by use of MG-63 osteosarcoma cell line. Their viability, proliferation and morphology in the fabricated scaffold were analyzed. In order to examine the osteogenic property of the scaffolds and their potential for bone regeneration, in vivo evaluation was done on osteoporosis rats with critical-size defect in their femoral site. Stereological analysis of histological parameters was conducted by evaluating total bone volume, cortical bone volume, trabecular bone volume, and total bone marrow volume. In addition, the number of osteocytes, osteoblasts, and osteoclasts were estimated and compared in studied groups.

## Materials and methods

### Materials

In this work Elastin from bovine neck ligament, (PCL) MW = 80000 Da, and nHA, were purchased from Sigma Aldrich. Acetic acid, formic acid and 1,1,1,3,3,3-hexafluoro-2- propanol were purchased from Merck (Germany).

### Scaffold fabrication method

**Fig. 1:**
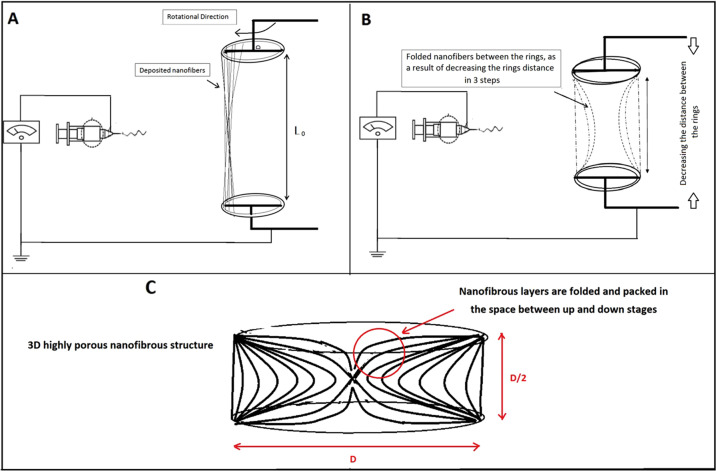
**A**–**C** Scheme of the scaffold fabrication procedure

In this work, a novel procedure was used for scaffold fabrication. The considered scaffold possessed a multilayer highly porous nanofibrous structure composed of elastin/PCL/nHA. In order to construct the mentioned multi-component scaffold, two electrospinning solutions containing PCL/nHA and PCL/elastin were prepared. For the preparation of PCL/nHA solution, 0.125 g PCL was dissolved in 1 ml of acetic acid/formic acid (9:1 v/v ratio) solvent system for 20 h under constant magnetic stirring in 40 °C, and 5 h prior to electrospinning 0.025 g nHA was added to the already prepared solution. In order to the preparation of PCL/elastin solution, 0.08 g PCL was dissolved in 1 ml of HFIP solvent. Subsequently, 0.02 g elastin was added to the system and the content was allowed to dissolve overnight in 40 °C under magnetic stirring. Each of the above solutions was poured in a 5 ml glass syringe and loaded in the electrospinning apparatus (Fanavaran nano-meghyas, Iran) in order to perform a co-electrospinning procedure (Fig. 1). Electrospinning was done with an applied voltage of 18 kV, flow rate of 0.8 ml/h and the needle tip to collector distance (air gap) of 10 cm for both PCL/elastin and PCL/nHA solutions. Electrospinning technique was applied by use of a new collector design. The design of the collector is as following:

The collector is composed of two negatively charged rotating rings (*D* = 1 cm). They were justified in a specific position and distance toward the spinning nozzle such that the nanofibers would be deposited between them. In the other word, nanofibers crossed the space between the two rings (Fig. 1A, B). Varying parameter during the procedure was the distance between the two rings, between *L*_0_ = 5*D* (at the beginning) to *D*/2=0.5 cm (at the end). The rings rotated at the same speed of about 10 rpm.As time went on, every 5 min the “*n*” value increased such that the distance between the two rings decreased as *L* = *L*0 − *n*(*D*/2). The “*n*” parameter was between 0 (at the beginning) to 9 (at the end) during the procedure. As a result, in each step a new separated highly porous nanofibrous layer was produced and folded between the rings gap as they approach. For example, after 15 min the structure would be as Fig. 1C. The rotation of collector rings provides the uniform collection of PCL/elatin and PCL/nHA as-spun fibers from each nozzle.

### SEM observation

Morphology of the prepared structures was evaluated with Scanning Electron Microscope (SEM, VEGA, TESCAN, Czech). Prior to the SEM imaging, surface of the samples was coated with a thin layer of gold (sputter coater, BAL TECH). Image analysis software (Image J™, NIH, MD, USA) was applied for determining the average diameter of fibers. For this aim, 25 fibers in each SEM image were considered and their diameters were measured. Average diameters are expressed as mean value ± standard deviation.

### Scaffold water uptake capacity

Water uptake capacity is an important parameter which guarantees the water and nutrients delivery to the regenerating regions. In this work, water uptake capacity of the prepared scaffolds was determined by using phosphate buffered saline solution (pH 7.4). After being weighted in dry condition (*w*_d_), scaffolds were soaked in PBS solution at 37 °C for 0.5, 1, 6, and 24 h and were weighted again (*w*_w_). The ratio of the weight increase (*w*_w_ − *w*_d_) to the initial weight (*w*_d_) at each time point, that was defined as the water uptake capacity. Five replicates were considered for each sample at each time interval and the water uptake capacity value was expressed as the means ± standard deviation.

### In vitro cytotoxicity

Indirect cytotoxicity test was done with Alamar Blue assay and using MG-63 cell line. Fabricated scaffolds were sterilized by exposure to Gama irradiant (25 KiloGrays) for 120 min and were immersed in Dulbecco’s Modified Eagle Medium (DMEM) medium supplemented with 10 % fetal bovine serum (FBS) and 1% penicillin/streptomycin to obtain the extracts. After 1 and 4 days, the extracts were removed and placed in the medium of pre-seeded cells which were seeded (density = 2 × 10^4^ cells/well) in a 96-well tissue culture plate for 24 h. Three replicates were considered for each eluate. After 24 h of incubation, 150 μl Alamar Blue^*TM*^ solution (10 % v/v in culture medium) was replaced the culture medium of each well and the plate incubated for another 4 h. 100 μl from the solution of each well was transferred to a 96 well plate and the absorbance in the wavelength of 540 nm was measured using Thermo Scientific Multiscan Spectrum plate reader (reference wavelength: 630 nm). Results reported as the relative ratio of sample values to the control ones (results of the non-interfered medium).

### In vitro cytocompatibilty

In vitro cytocompatibility of the scaffolds was assessed using the MG63 cell line. After being sterilized by exposure to Gama irradiant for 120 min, scaffolds were placed in 48-multiwell culture plate, seeded with cell suspension (4 × 10^4^ cells/well) and cultured in an incubator up to 14 days. At considered time points, cells viability was assessed by replacing culture medium with 1 ml Alamar Blue^TM^ solution (10% v/v in culture medium). After being incubated for 4 h, 100 μl of the solution, in triplicate, was removed from each well, transferred to a 96 well plate and the absorbance in the wavelength of 540 nm was measured using Thermo Scientific Multiscan Spectrum plate reader (reference wavelength: 630 nm). Samples were subsequently rinsed with PBS and fresh culture medium was added to each well and the plate was returned to the incubator until the next time-point.

### Cell morphology

In order to evaluate the morphology of the cells cultured on the scaffolds, samples were seeded with MG-63 osteosarcoma cell line (4 × 10^4^ cells/sample) and after 4 and 7 days, were fixed with 4% glutaraldehyde solution for 30 min. After rinsing with PBS buffer and dehydration in ethanol solutions, samples were dried in a vacuum overnight and observed by scanning electron microscope (Philips XL30 SEM, Korea).

### Isolation of bone marrow mesenchymal stem cells (BM-MSCs) and cell culture

BM-MSCs were isolated from 8-week-old female rats. Rats were sacrificed, the femora were dissected out under sterile conditions, and the edge of each bone was cut. DMEM (Gibco, USA) was then injected into the bone marrow using an 18-gauge syringe, and the bone marrow cells were flushed out to the opposite side; this procedure was repeated several times. Afterwards, the bone marrow cells were seeded into a tissue culture flask in DMEM containing an antibiotic-antimitotic solution (100 units/ml penicillin G and 100 mg/ml streptomycin, both from Gibco, USA), and the medium was supplemented with 10% FBS. Three days after seeding, floating cells were removed, and the medium was replaced with a fresh medium. The adherent, spindle-shaped cells were passaged when the cells approached confluence. Adherent cells were collected with Trypsin/Ethylenediaminetetraacetic acid (EDTA), re-suspended in a fresh medium and transferred to new flasks at a density of 1 × 104 cells/cm^2^. The fixed cells were washed twice with PBS (Sigma, USA) and incubated at 4 °C with antibodies to the following antigens: CD34, CD45, CD90, and CD44 (all from Chemicon, USA) for 30 min. Primary antibodies were directly conjugated with phycoerythrinphycoerythrin. Flow cytometry was performed with a FACS can flow cytometer (Becton Dickinson, USA), and flow cytometry analysis was carried out by a PartecCyFlow Space cytometer using FloMax software.

### Osteogenic differentiation of BM-MSCs on the 3D scaffold

Osteogenic differentiation of BM-MSCs performed on the 3D scaffold of 5 mm diameters and 2-3 mm heights in vitro. 3D Scaffold was placed in 24-well culture plates, washed 3 times with 70% ethanol, exposure to U.V for an hour and then washed twice with osteogenic medium. BM-MSCs were seeded on the 3D scaffold at a density of 10 × 10^6^ and incubated at 37 °C in osteogenic differentiation medium containing low-glucose DMEM containing 10% FBS, 0.1 μM dexamethasone (Sigma Aldrich, USA), 200 μM L-ascorbic acid-2-phosphate (Sigma-Aldrich, USA), and 10 mM β-glycerol phosphate (Sigma-Aldrich, USA) for three weeks. The scaffold carrying osteogenic differentiated BM-MSCs cells was fixed at 21st day using Glutaraldehyde and analysis by SEM and then applied to the induced femoral defect in the rat. Osteogenic induction was confirmed by Alizarin Red S staining.

### Animals

In total, 24 female Wistar rats with mean weight of 200 ± 20 g and 8-week old were obtained from laboratory animal center of Shahid Beheshti University of Medical Sciences, Tehran, Iran. Animals were randomly divided into three groups: Group I: control+ bone defect, Group II: scaffold+ bone defect and Group III: scaffold+ bone defect +cell. Each group included eight rats which were housed in 4 under standard conditions (room temperature and 12:12 h light-dark schedule) and had free access to water and food.

### OVX procedure

All rats underwent total OVX. At the surgery day, the rats were anesthetized by ketamine (50 mg/kg) and xylazine (5 mg/kg). Two paravertebral skin incisions were done and ligated the uterine tubes. After the ovaries were removed, we sutured the incisions. Each rat received ceftriaxone (50 mg/kg, Jaberben Hayan, Iran) injections, as antibiotic therapy, before the surgery, 24 and 48 h after surgery. All animals were kept for 3 months after surgery in cages to develop OP. At the end of this period, the rats were submitted to CT scanning for confirmation of OP.

### CT scanning

**Fig. 2:**
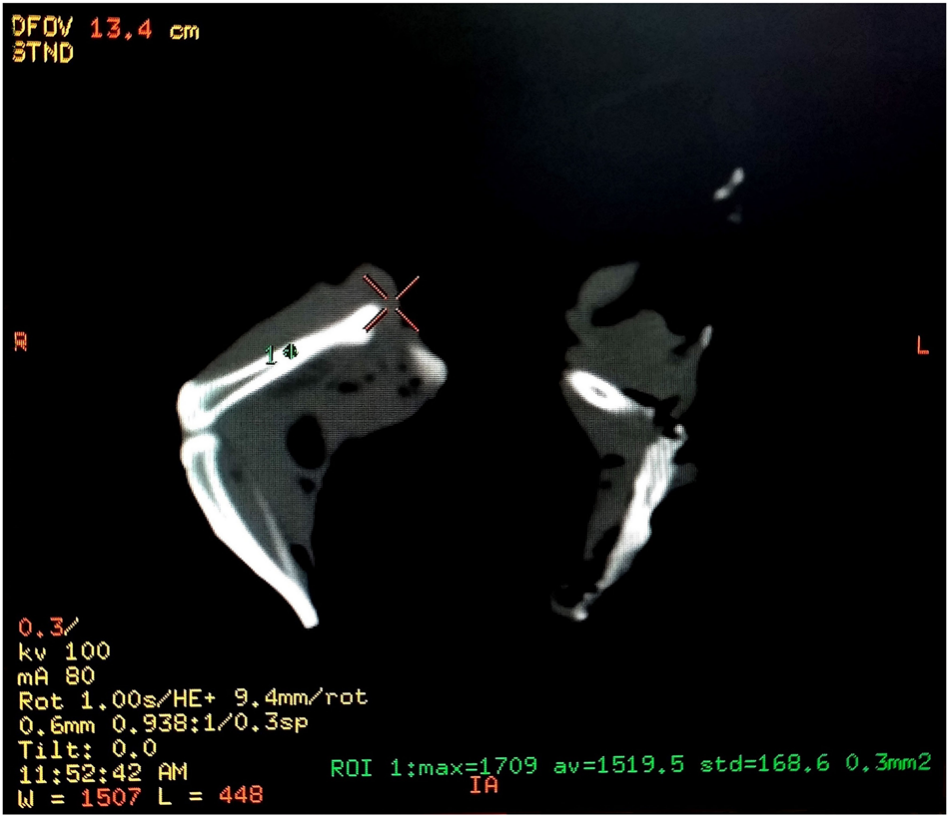
Computed tomography scan (CT scan) from the femur of a rat. Bone densities were measured in Hounsfield Unit (HU)

First OP development of OVX rat femur were evaluated qualitatively by CT scan using multi slides (kv = 100, Ma = 50, sections=2 mm, FOV=240 mm; GE, 16 Slice, USA) compared with healthy rats 3 months after OVX. Femur bone density was determined while the rats were under general anesthesia. Bone mineral densities were reported in HU. The technician was unaware of the study group assignments (Table 1 and Fig. 2).

**Table 1:** Mean ± SD of HU in the control and OVX groups

Groups CT number	Control	OVX
Hounsfield Unit (HU)	870 ± 34	560 ± 26

### Partial osteotomy

Osteotomy was performed under complete anesthesia (30 mg/kg ketamine, 3 mg/kg xylazine). Briefly, the skin of the right leg of each rat was cut longitudinally to expose the Femur midshaft. A partial osteotomy was created with a low-speed drill (terminal, 3 mm diameter; Delab; Dental Fabriktreffurt, Germany).

### Tissue preparation

Histological evaluation was performed at 4 weeks after surgery. Every femur was removed, and soft tissues including skins and muscles were eliminated from the femur. Tissue samples (proximal half of each right femur including the fractured and defected areas) were fixed in 10% formalin for 48 h and decalcified in 10% nitric acid. Then the defected areas were embedded in paraffin blocks and cut longitudinally into 5 μm and 25 μm thick sections with a microtome. For the microscopic descriptive analysis of each group, slides were stained by hematoxylin and eosin (H&E) and Masson’s trichrome dyes. Bone healing evaluation was performed using a microscope connected to an image analyzer.

### Stereological study

The bone volume was measured using a microscope connected to a camera; volumes of bone tissue, volumes of trabecular bone and bone marrow were calculated using the Cavalieri method [21] as the product of the areas and measured tissue thickness between the saved sections. Using stereological software, the total area of the sections (∑*A*) of the femur fracture region was determined, and finally, the volume was estimated by the following formulation:

$$V = {\sum} {P \times \frac{a}{p} \times t}$$

where “Σ*P*” is the total points hitting the tissue sections, “a/p” is the area associated with each point, and “*t*” is the distance between the sampled sections.

### Measurement of total number of the bone cells

For the estimation of numerical density and total number of the osteocyte, osteoblast and osteoclast, dissector method was used. Sections were measured with the optical dissector [21]. The specimens were evaluated at ×40 oil immersion magnification with a high numerical aperture. The image was captured and analyzed by a computer. The focus plane was set at the surface of the specimen. Then a set of three unbiased measurement frames was superimposed on the live image. At the same time, the microcator measuring the optical distance through the specimen in the z axis was zeroed. By gently moving the focus down through the specimen, an approximately 0.5-mm thin focal plane made objects come into focus and disappear. Bone cells falling in the measurement frames’ permitted areas were counted as they came into focus until the microcator indicated that the focal plane had traveled 10 µm through the specimen. The numerical density of cells was obtained by the following formulation:

$$N_v = \frac{{{\sum} Q }}{{{\sum} {P \times h \times \frac{a}{f}} }} \times \frac{t}{{BA}}$$

where Σ*Q*-” is the number of the nuclei coming into focus and counted, “Σ*P*” is the total number of the counting frames in all fields, “a/f” is the area per frame, “*h*” is the height of the disector, “*t*” is the real section thickness measured using the microcator when the *Q*- was counted, and “BA” was the block advance of the microtome. To estimate the total number of the bone cells, the following formula was used: *N* (bone cell) = Nv × *V*.

### Real-time PCR

The total RNA samples extracted and treated with DNase I (Roche, Basel, Switzerland) to remove genomic DNA contamination. cDNA was synthesized in a total volume of 20 μl using a commercial kit (Fermentas, Lithuania) at 42 °C for 60 min. The Applied Real-time PCR (TaqMan) was used according to QuantiTect SYBR Green RT-PCR kit Takara Bio Inc, Japan) for quantification of mRNA expression levels of BMP-2 and VGEF between different groups. All studied forward and reverse primer pairs was designed according to Primer 3 Plus software in exon-exon junction way to distinguish between cDNA and genomic DNA. Formerly, the PCR primers were tested by Primer-Blast tool at the site, www.ncbi.nlm.nih.gov/tools/primer-blast (Table 2).

**Tab 2:** Primers design

Genes (Accession number)	Primer sequences
GAPDH	F = CCTTCCGTGTTCCTACCC R = CAACCTGGTCCTCAGTGTAG
BMP-2	F = GGAACATAAGGCACGCTGAAC R = 5′TGAGGAAGCAGGTAGATGGTGA
VEGF	F = CTGTGCAGGCTGCTGTAACG R = GTTCCCGAAACCCTGAGGAG

### Statistical analysis

Data were analyzed using Kruskall–Wallis and Mann–Whitney *U*-test with adjusted alpha level. *P* ≤ 0.05 was considered as statistical significance.

## Results

### Structural properties

**Fig. 3:**
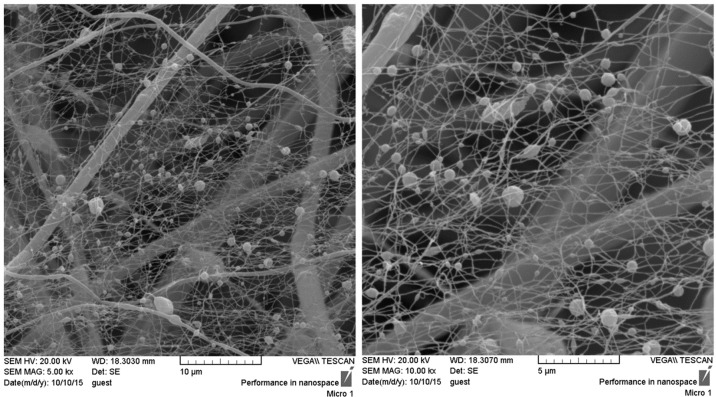
Scanning electron microscope images representing the microstructure of 3D nanofibrous PCL/elastin/nHA scaffold

Microstructure of the fabricated scaffolds is shown in Fig. 3. The obtained porous structure with non-compact position of nanofibers compared with the compact 2D nanofibrous mat which is routinely seen in conventional electrospinning confirms the success of the applied collector in the fabrication of 3D highly porous nanofibrous scaffold. The obtained scaffolds with non-compact structure of the nanofibers would provide favorable condition for cellular infiltration and three-dimensional tissue regeneration. According to the SEM images, the fabricated structure exhibited two populations of fiber diameters size, one population in the nanometer range with an average of 117.6 ± 52.88 nm and the other in the micrometer range with an average of 1.7 ± 0.8 μm.

### Water uptake capacity

**Fig. 4:**
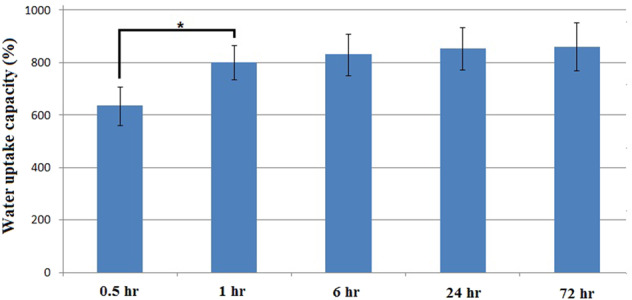
Water uptake capacity of the fabricated scaffolds. Significant difference is denoted as (*p < 0.05). Error bars represent the standard deviation of five independent measurements

Figure 4 represents water uptake capacity of the fabricated scaffolds. Observation indicates that water was immediately absorbed into the modified nanofibrous structure such that water uptake value of about 600% was obtained after just half an hour. One hour after being immersed in PBS solution water uptake reached the value of 800% and a slight increase up to 830% was seen after 6 h. Afterwards no significant difference was observed between the values of the following time points indicating that the scaffolds were saturated with water within the first hours of immersion.

### Indirect cytotoxicity evaluation

Prior to in vivo implantation, indirect cytotoxicity test using MG-63 cell line and Alamar Blue assay was applied to evaluate the probable cytotoxic effect of the prepared scaffolds. Results indicated the complete cell viability for the fabricated scaffolds extracts (Table 3).

**Table 3:** In vitro cell viability at the exposure to the scaffold extracts

Cell viability (%) Sample	Day 1	Day 4
Elastin/PCL/nHA scaffold	97.25 ± 8.70	96.82 ± 10.59

### Direct cytocompatibility evaluation

**Fig. 5:**
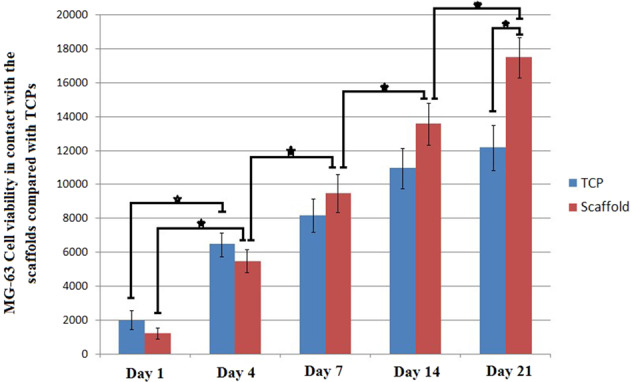
MG-63 cell viability in contact with the prepare scaffolds compared to TCPS at the considered time points. Significant difference is denoted as * (*p* < 0.05). Error bars represent the standard deviation of three independent measurements

Quantitative results of the direct cytocompatibility evaluation are reported in Fig. 5. According to the data, as the cells were seeded on the fabricated scaffolds their proliferation rate was significantly increased compared with TCPs, although the value of cell viability on the TCPs was higher at the initial time points. Furthermore, although the cell proliferation on TCPs revealed no significant increase (with one exception between 1st and 4th days), cell proliferation on the fabricated scaffolds lasts for a long duration in such a way that significant difference between each two subsequent time points was observed until the end of experiment.

### Cellular morphology

**Fig. 6:**
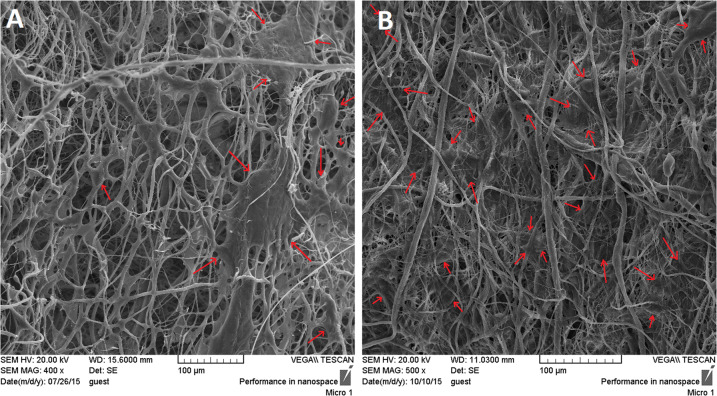
**A** and **B**: Morphology of the MG-63 cells seeded on the fabricated scaffolds after 4 and 7 days of culture, respectively

SEM images of MG63 cells seeded on the 3D nanofibrous scaffold are represented in Fig. 6. Arrows show the cell populations on the nanofibrous structure. As time goes on (comparing the images of 4 and 7 days after cell seeding), due to the proper cell proliferation the entire region is filled with cells to the extent that no free space can be seen between the nanofibers at day 7. The obtained results indicate that the fabricated scaffold can provide favorable condition for cells attachment and proliferation.

### Rat BMSCs characterization

**Fig. 7:**
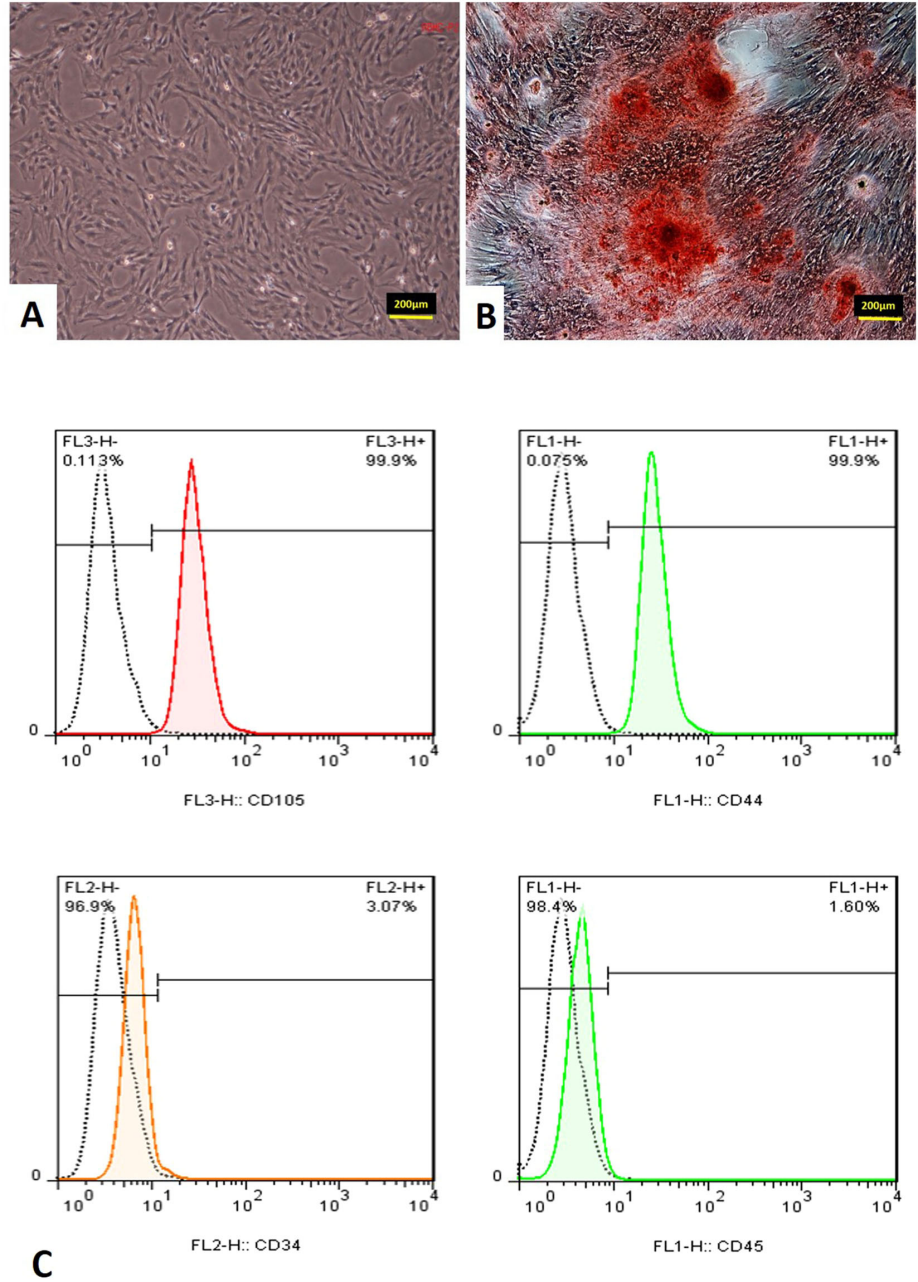
**A** Mesenchymal stem cells of the fourth passage. **B** Osteogenic differentiation by Alizarin Red S staining for mineral deposition was performed. **C** Flow cytometry analysis of passage three mesenchymal stem cells culture for CD105, CD44, CD34, and CD45 cells

The BMSCs appeared as a monolayer of large, fibroblast-like flattened adherent cells at passage 4 (Fig. 7A). Then the osteogenic induction was confirmed by Alizarin Red S staining (Fig. 7B). Flow cytometry analysis of rat BMSCs within 3 passages showed that rat BMSCs with elevated expressions of the characteristic MSC surface markers CD105 (99.9%) and CD44 (99.9%), and decreased expressions of hematopoietic markers CD34 (3.07%) and CD45 (1.60%) (Fig. 7C).

## Stereological results

### Total volumes of the bone, trabecular bone and bone marrow

**Fig. 8:**
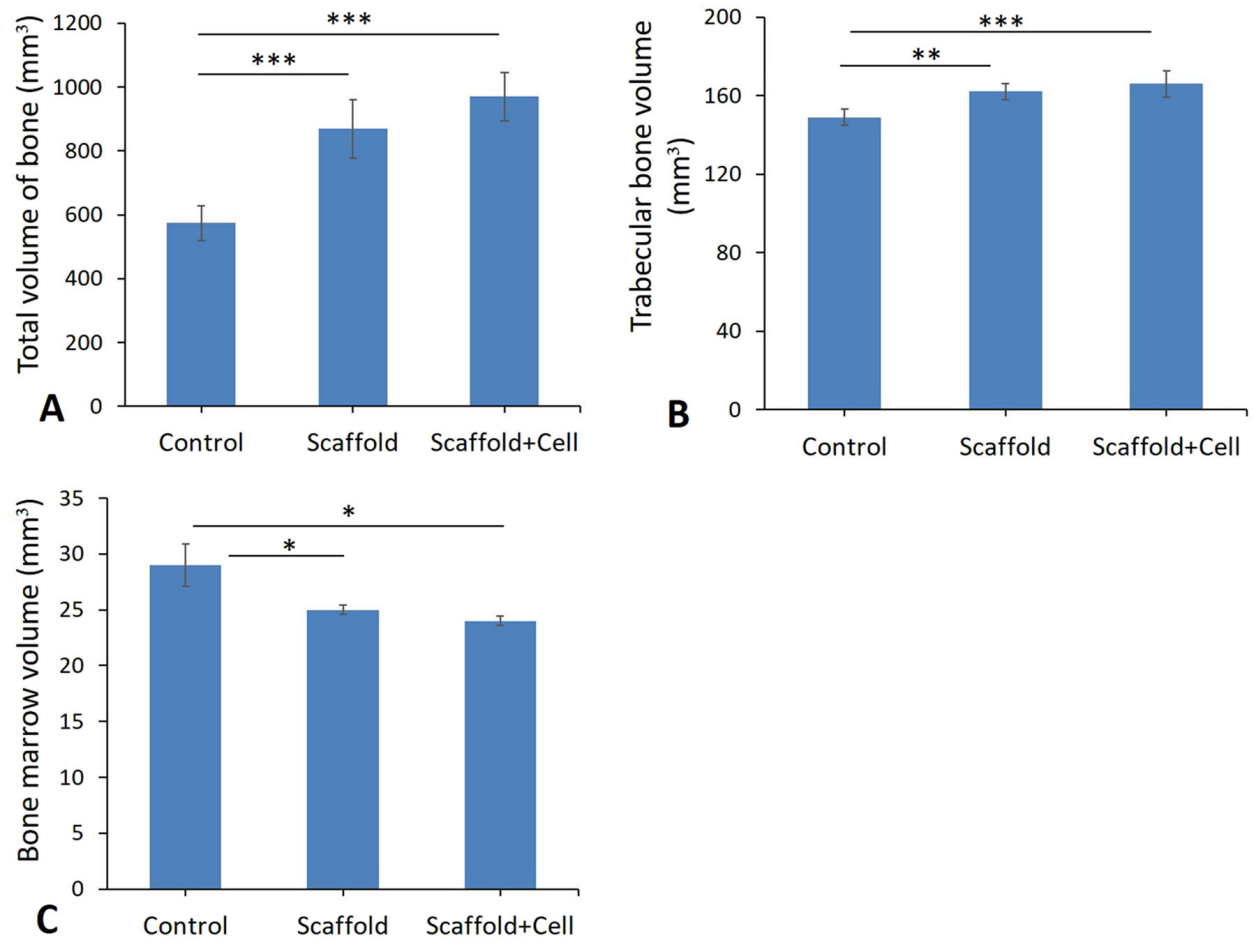
Mean ± SEM of total volume of bone (**A**), trabecular bone volume (**B**) and bone marrow volume (**C**), in studied groups compared by One-Way ANOVA test; (*p < 0.05, **p < 0.01, ***p < 0.001)

**Fig. 9:**
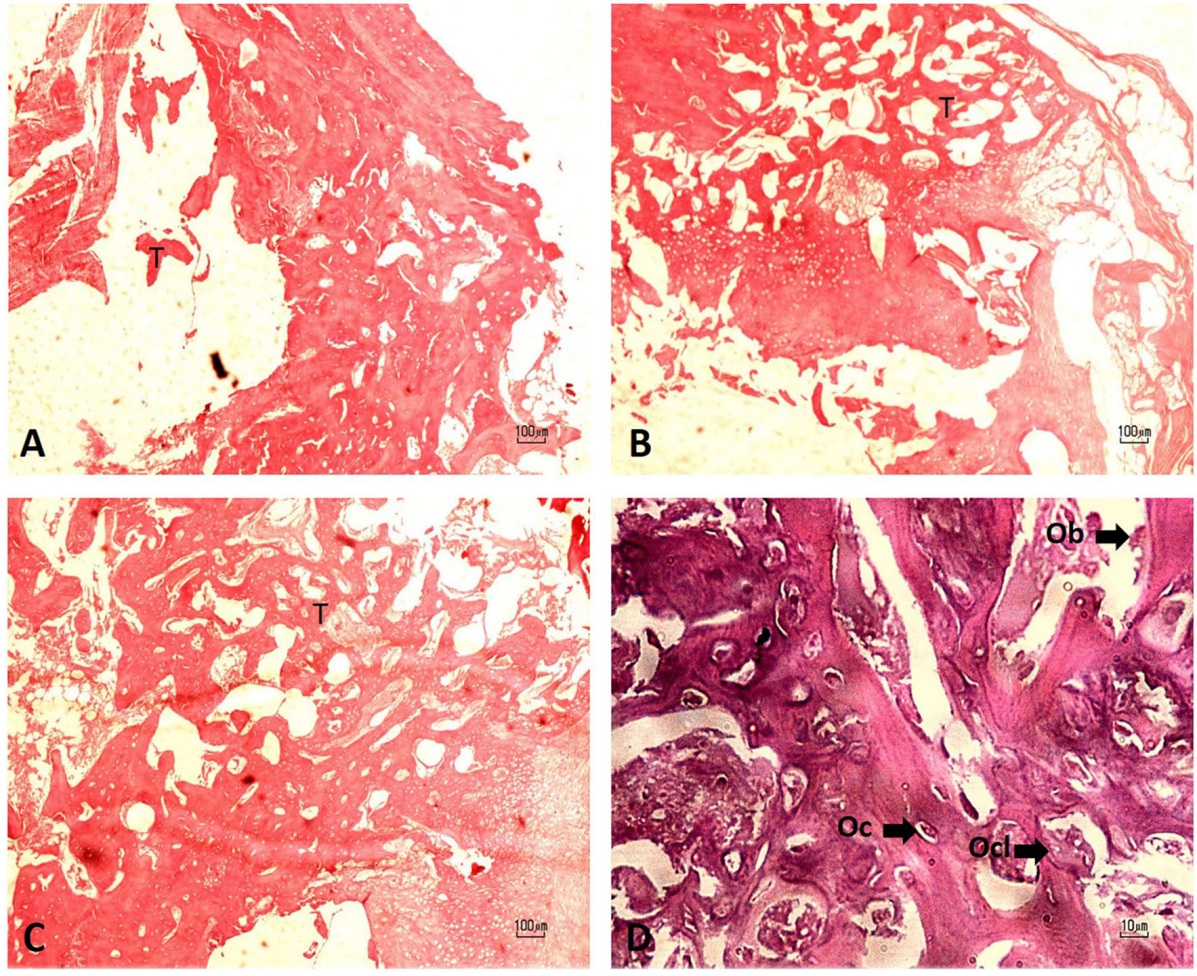
Micrograph of the bone defect stained with H& E. **A** Control; **B** Scaffold; **C** Scaffold+cell, Trabecular bone (T). **D** octeoblast (Ob), ocsteocyte (Oc), and osteoclast (Ocl)

Based on the findings of the current study, total volumes of the bone, trabecular bone and bone marrow reduction in the control groups compared to the scaffold and scaffold+cell groups (Fig. 8, A–C). The data revealed that the use of the scaffold and MSC has effect on total volumes of the bone, trabecular bone and bone marrow at the osteotomy site in osteoporosis rat models (Fig. 8, A–C and Fig. 9 A–D).

### Total numbers of osteoblasts, osteocytes, and osteoclasts

**Fig. 10:**
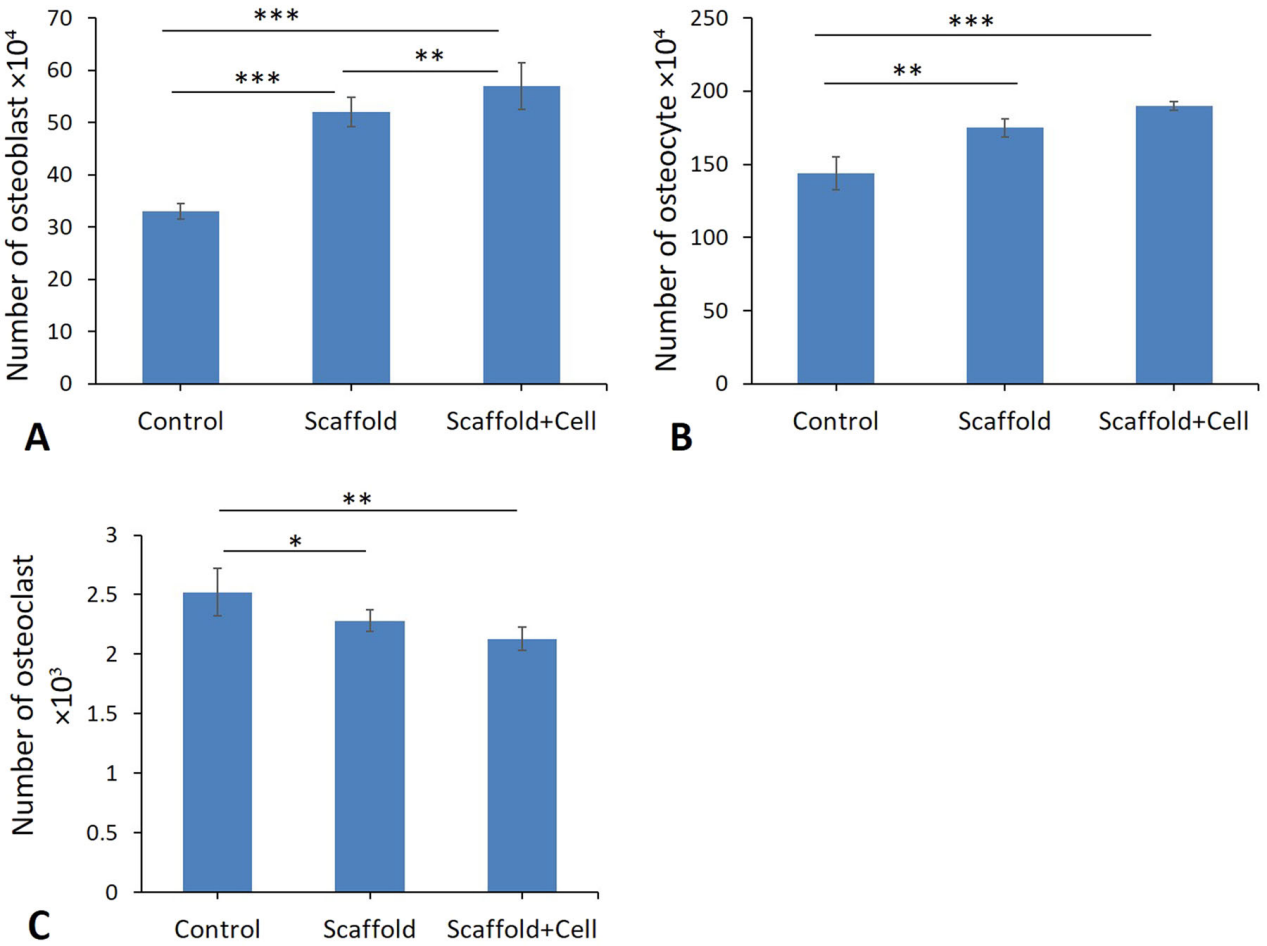
Mean ± SEM of total number of osteoblast (A), osteocyte (B) and osteoclast (C), in studied groups compared by One-Way ANOVA test; (*p < 0.05, **p < 0.01, ***p < 0.001)

The results showed a significant decrease in the total number of the osteoblast, osteocyte and increase in osteoclast of bone at the osteotomy site of the control group in comparison to the scaffold and scaffold + cells groups (*p* < 0.02) (Fig. 10A–C). However, study of the data showed that use of the scaffold and MSC increases the total number of the osteocyte, osteoblast and osteoclast in the scaffold and scaffold in combination with MSC increases the total number of the osteocyte, osteoblast and decrease the total number of osteoclast compared to the control group (Fig. 9A–D and Fig. 10A–C).

### Expression levels in Bmp-2 and Vegf using real-time PCR

**Fig. 11:**
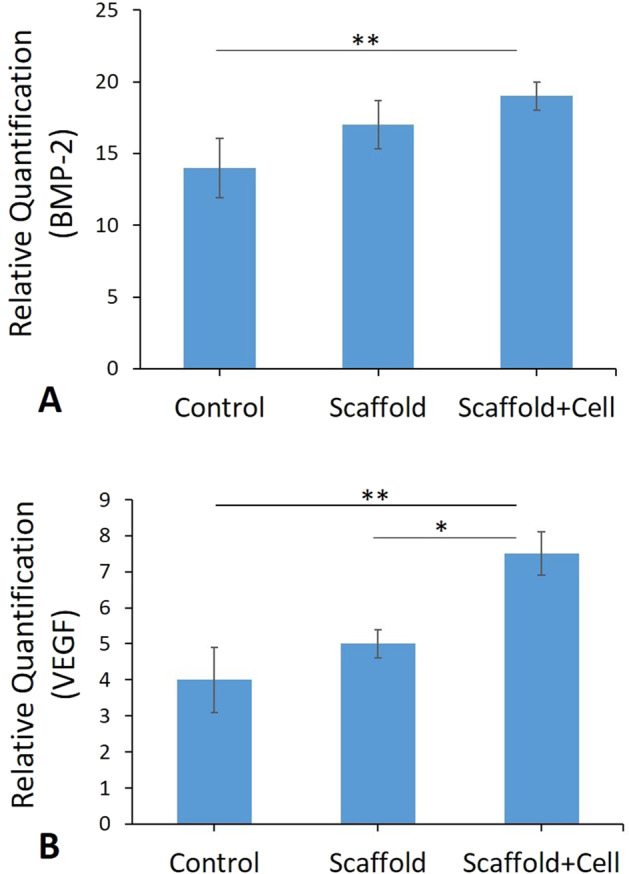
(**A** and **B**) Real-time -PCR analyses of testes. mRNA expression levels of BMP-2 and VEGF from Control; Scaffold; Scaffold+cell groups. (**p < 0.01)

Relative levels of mRNA expression in *Bmp-2* and *Vegf* were normalized and quantified in various groups. As depicted in Fig. 11A, the levels of *Bmp-2* expression enhanced remarkably in the scaffold + cells group compared to the control groups (*P* < 0.01). Nonetheless, no significant differences were observed between scaffold + cells group and scaffold groups (Fig. 11A). Outputs also reflected significant enhancement in expression levels of *Vegf* in the scaffold + cells group compared to the scaffold group (*P* < 0.05) and control group (*P* < 0.01) (Fig. 11 B).

## Discussion

Each year, thousands of people in the world suffer from various kinds of bone disease and defects such as trauma or tumors. On the other hand, many people die due to insufficient perfect bone substitute [4]. Besides, there is an extensive history of using auto graft or allograft bones for treatment of bone defects. Several novel attitudes have been established for the construction of three-dimensional biomaterial scaffolds [22].

Features of biomaterials are morphologically similar to extracellular matrix (ECM) of normal cells. Accordingly, many specialists and researchers have considered the usage of inorganic material-based polymer composites as scaffolds for tissue reconstructing [23].

Natural biological macromolecules such as proteins extracted from bone, cartilage, ligament, skin or other tissues of living bodies provide proper condition for cellular growth and tissue regeneration [24, 25]. Tropoelastin, the principal component of elastin, consists of mostly non-polar amino acids arranged in alternating hydrophobic and hydrophilic domains [1]. This biologically active molecule mediates cellular processes such as cytoskeletal organization, chemotaxis, proliferation, and differentiation. Furthermore, presence of this biological macromolecule, interfere the local tissue environment by regulating the matrix proteases [26].

Mixing natural biomaterials with different synthetic nanomaterials such as nHA, Carbon nanotubes (CNT) and polymers such as polycaprolactone (PCL), called polymeric nano biomaterials, has been approved by other researchers [17, 27, 28]. This method is a practical tool to decrease adverse effects like cytotoxicity, following chemical cross-linking substances usage [17, 29].

In this study the application of an innovative scaffold possessing highly porous 3D nanofibrous structure composed of PCL/elastin/nHA was investigated as a promising scaffold for bone tissue engineering. A novel collector design was introduced for the fabrication of nanofibrous structure by electrospinning technique which results in the special properties including enhancement of porosity and pore size, reducing the packing density of nanofibers in the structure, and providing three-dimensional scaffold which is suitable for critical size tissue defects. Along with the proper microstructure, applying elastin as a highly potent natural macromolecule and nanohydroxyapatite as an osteogenic biomolecule in addition to polycaprolactone as basement for the scaffold fabrication provided favorable condition for bone tissue regeneration. According to the morphological observations, a highly porous nanofibrous structure is produced which consists of a combination of micro and nano-sizefibers.

According to the literature, PCL/elasin nanofibers (produced by the similar electrospinning parameters and electrospinning solution) possess smaller diameter compared with PCL/nHA fibers [30, 31]. Therefor, the majority of the nanofibers with average diameter about 100 nm presented in the SEM images would have PCL/elastin composition. By the way, perturbations in the PCL/nHA electrospinning solution may result in branching some of the main PCL/nHA fibers into nanofibers with smaller diameters because of the existence of nHA nanoparticles. In addition, inconsistency in the electrical field due to the special design of the collector which provides conductive-nonconductive regions may also result in perturbations and the subsequent bimodal distribution of fiber diameters.

Beside the effect of micro-size pores formed by the microfibers on the enhancement of cellular migration, small pores formed between nano-size fibers is proven to have positive effect on cell proliferation, adhesion, and differentiation into osteoblasts in vitro and stimulating new bone formation in osseous defect model in vivo by providing suitable environment for cellular activity [32].

Because of the three dimentional and highly porous feature of the fabricated nanofibrous structure (compared with the compact structure of conventional 2D electrospun mat) which provides the vast available free space for liquid to permeate, a high value of water absorption (about 800%) is observed. Although as the result of hydrophilic nature and polar functional groups of elastin and nHA adsorption of water molecules and mineral elements on the surface of fibers may also occur, the absorption of fluid into the vast free space within the sample would be responsible for the high value of water uptake. By the way, existence of elastin and nHA would accelerate water diffusion and reduce the saturation duration such that after just a few hours no significant difference was observed between the water uptake values of the subsequent time points. High value and short duration of water uptake guarantees the nutrient diffusion and body fluid exchange between the scaffold and biological environment. According to the in vitro cytocompatibility results, cell proliferation on the scaffolds was intensified and lasts for long time compared with the cells on TCPs, as they have vast free space for growth within the provided highly porous nanofibrous structure.

Using stereological-based methods, the present study assessed the bone defect volume, trabecular bone volume, bone marrow volume, and the total number of bone cells including osteoblasts, osteocytes, and osteoclasts after cell-based therapy. Overall outcomes of this study revealed encouraging impacts of simultaneous use of mesenchymal stem cells (MSCs) and three-dimensional nanofibrous scaffold comprising elastin/pcl/nHA in an experimental animal model of bone defect.

In the present study the transplantation of MSCs on the considered scaffold was used assess cell survival and bone healing. As an effective cellular source, MSCs were selected since several experimental and clinical studies have shown potential of these stem cells to cure various tissue defects such as bone defects. In addition, it has been found that MSCs were successfully differentiated into osteoblasts and start new bone matrix formation. MSCs produce some growth factors like VEGF, FGF-2, TGF-β, BMP-2 [8, 9]. Some of these growth factors such as *Bmp-2* and *Vegf* are required for differentiation and proliferation of osteoblast and bone formation [8–10]. This survey revealed improvements in the stereological parameters and also in expression of *Bmp-2* and *Vegf* genes in the rat scaffold in combination with mesenchymal stem cells in comparison with the osteoporotic rat models. In vitro results also indicated significant increase in trabecular bone volume, bone marrow volume, total number of osteoblasts, osteocytes, and osteoblast in scaffold+ cell group compared to the both control and scaffold groups. Moreover, the increased level of *Bmp-2* and *Vegf* genes expression in the scaffold+ cell group in comparison with the other groups was observed.

MSCs culture results have disclosed that the incorporation of the fabricated nanofibrous scaffold was able to enhance cell capability to osteogenic proliferation and differentiation [17].

MSCs were used in present work owing to the potential capabilities to provide more clinically important evidence from our studies. It was previously described that MSCs seeding on the scaffold materials had less spread and more proliferation and differentiation rate than MSCs on defect area without scaffold [33–35]. Hence, the results presented that concurrent use of the fabricated scaffold and cell is capable to improve histological parameters in bone defect areas.

## Conclusion

In the present study, a novel composition, which has not been reported previously, is considered so that the highly potent scaffold would be obtained and an innovative method is introduced for the fabrication of highly porous nanofibrous scaffolds for 3D defects. In addition, OVX model is considered in order to evaluate the success of tissue engineering method in abnormal conditions and pre-seeding of MSCs on the scaffolds is performed to intensify the bone tissue regeneration.

The results showed that the 3D Elastin/Polycaprolactone/nHA scaffold in combination with bone marrow mesenchymal stem cells could improve the bone repair process and accelerate the ossification process in an ovariectomized rat model of osteoporosis.

## Supplementary information

Supplementary Information
