# Supplementary figures and images for: Improved healing of critical-size femoral defect in osteoporosis rat models using 3D elastin/polycaprolactone/nHA scaffold in combination with mesenchymal stem cells
Source: J Mater Sci Mater Med. 2021 Mar 8;32(3):27. doi: 10.1007/s10856-021-06495-w (PMC7940275; doi:10.1007/s10856-021-06495-w)

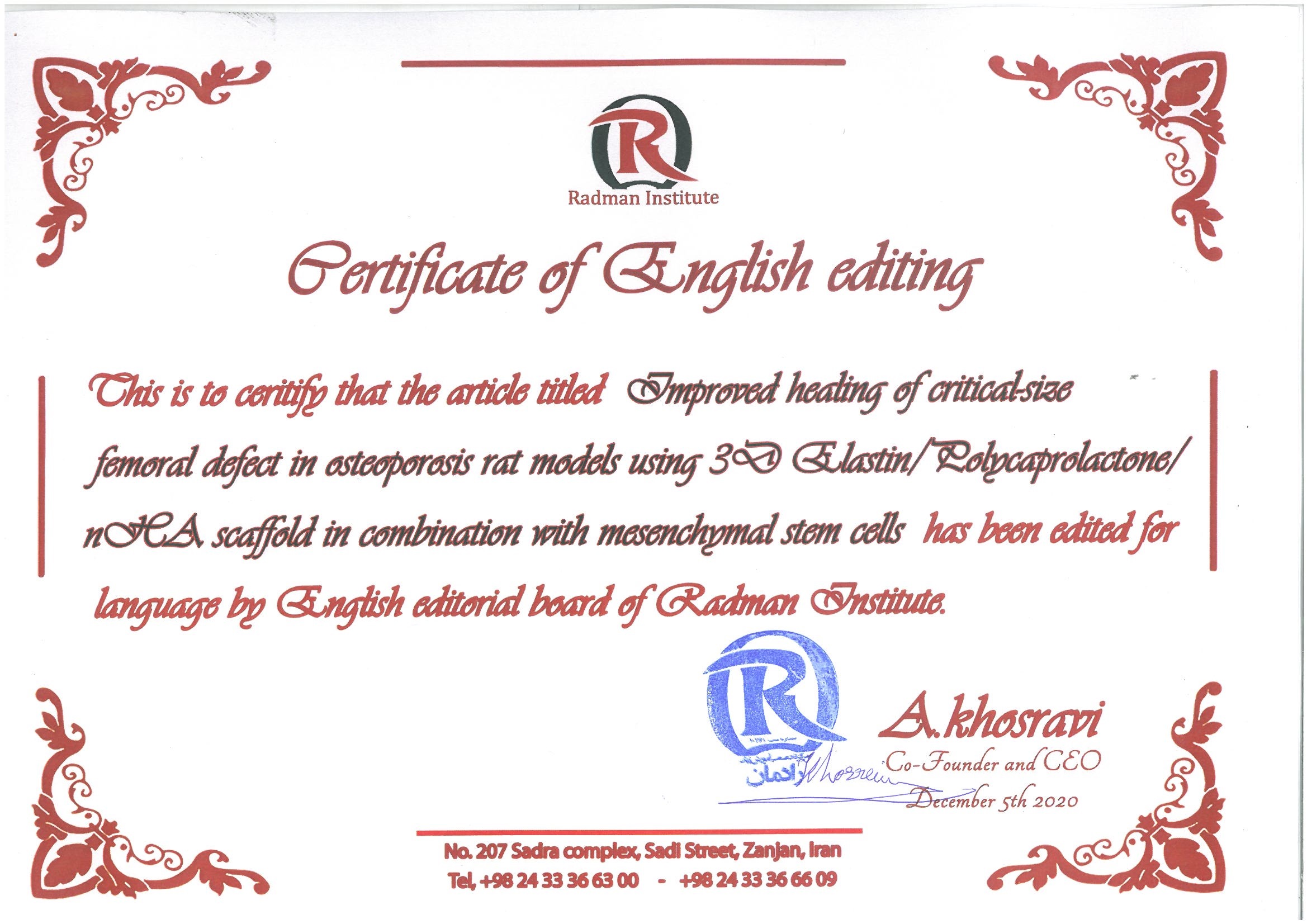

Supplement: Supplementary file 1 — Supplementary Information [file 10856_2021_6495_MOESM1_ESM.jpg]
